# Recent *vs* Conventional Methods of Caries Removal: A Comparative *in vivo* Study in Pediatric Patients

**DOI:** 10.5005/jp-journals-10005-1275

**Published:** 2015-04-28

**Authors:** Swati Chowdhry, Sonali Saha, Firoza Samadi, JN Jaiswal, Aarti Garg, Preet Chowdhry

**Affiliations:** Senior Lecturer, Department of Pedodontics and Preventive Dentistry, Manav Rachna Dental College, Faridabad, Haryana, India; Reader, Department of Pedodontics and Preventive Dentistry Sardar Patel Institute of Dental and Medical Sciences Lucknow, Uttar Pradesh, India; Professor, Department of Pedodontics and Preventive Dentistry, Career Post Graduate Institute of Dental Sciences and Hospital Lucknow, Uttar Pradesh, India; Professor and Director, Department of Pedodontics and Preventive Dentistry Sardar Patel Institute of Dental and Medical Sciences Lucknow, Uttar Pradesh, India; Senior Lecturer, Department of Pedodontics and Preventive Dentistry Sardar Patel Institute of Dental and Medical Sciences Lucknow, Uttar Pradesh, India; Vice Chairman, Department of Prosthodontics and Implantology, Shah Dental Care and Implant Centre, New Delhi, India

**Keywords:** Chemomechanical caries removal, Clinical efficacy, Microbiological assessment, Patient acceptability.

## Abstract

**Aims:** To compare the three different methods of caries removal, conventional method using Airotor and chemomechanical method using Carisolv and Papacarie.

**Settings and design:** The patients with multiple carious teeth were selected either in the deciduous dentition or mixed dentition. Ninety primary molars were selected from 30 children (10 males and 20 females) between the age group 6 and 9 years.

**Materials and methods:** After caries excavation, cavities were evaluated for caries removal or clinical efficacy by the tactile and visual criteria, microbiological efficacy, time taken for the procedure. Patient acceptability toward the treatment was also checked with the help of a visual analog scale (VAS). The observations thus obtained were subjected to statistical analysis using analysis of variance (ANOVA), Mann-Whitney U-test and Kruskal-Wallis test.

**Results:** The clinical efficacy of caries removal was highest with Airotor while the microbiological efficacy of caries removal was almost comparable with Airotor, Carisolv and Papacarie caries removal methods. The time taken to remove caries by Airotor method was observed to be least while the patient acceptance was found to be highest with Papacarie method.

**How to cite this article:** Chowdhry S, Saha S, Samadi F, Jaiswal JN, Garg A, Chowdhry P. Recent *vs* Conventional Methods of Caries Removal: A Comparative *in vivo* Study in Pediatric Patients. Int J Clin Pediatr Dent 2015;8(1):6-11.

## INTRODUCTION

Dental caries has inficted mankind from the very beginning and has encompassed every part of the globe thus justifying the wide spread of this pandemic disease.^1^ Once it affects the tooth structure, it is of fundamental importance to use conservative procedures that simultaneously prevent the progress of the lesion and minimize healthy tooth structure wear.^2^

Conventional caries removal and cavity preparation entail the use of high speed handpiece and burs which undoubtedly improved the speed and efficiency of cavity preparation but has many inevitable disadvantages, such as (i) perception of unpleasantness by the patients, (ii) use of local anesthesia, (iii) deleterious thermal effects, (iv) pressure effects on the pulp and (v) may result in removal of healthy dentin, resulting in an excessive loss of sound tooth structure.^3^

In quest to harness newer technologies for caries removal multifarious, new methods have been introduced. The chemomechanical caries removal has been introduced as an alternative noninvasive method of caries removal which aims at removal of infected tissues, together with eliminating the use of local anesthesia, avoiding pulp irritation with minimal or no patient discomfort.^4,5^

The essence is to summarize and highlight the need for further and profound research, to assess the efficacy of this caries removal method as compared with conventional method. Therefore, this study is undertaken to compare the clinical and microbiological efficacy, treatment time and patient acceptance of the conventional method of caries removal and chemomechanical caries removal using Carisolv and Papacarie.

## MATERIALS AND METHODS

**Fig. 1:**
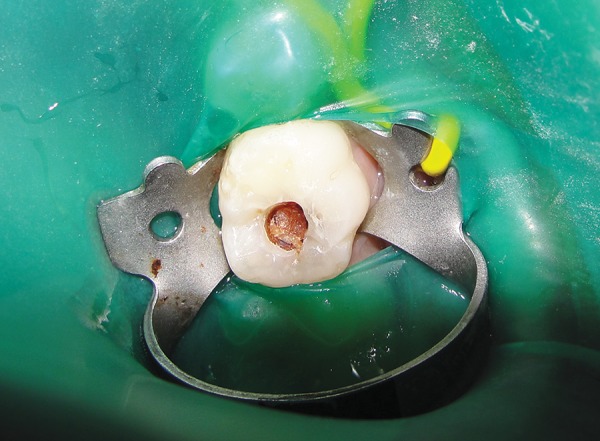
Preoperative photograph showing caries

**Fig. 2:**
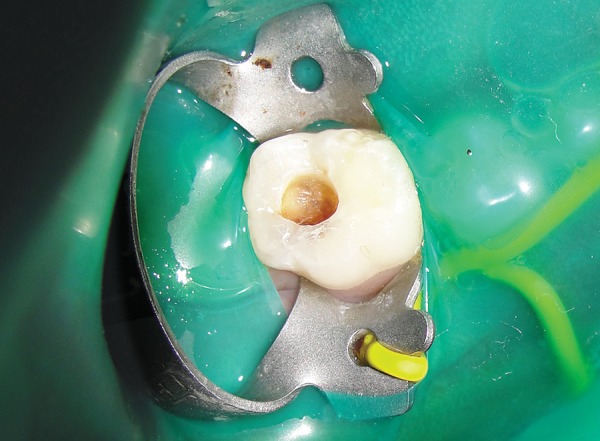
Postoperative photograph showing caries removal using Airotor

**Fig. 3:**
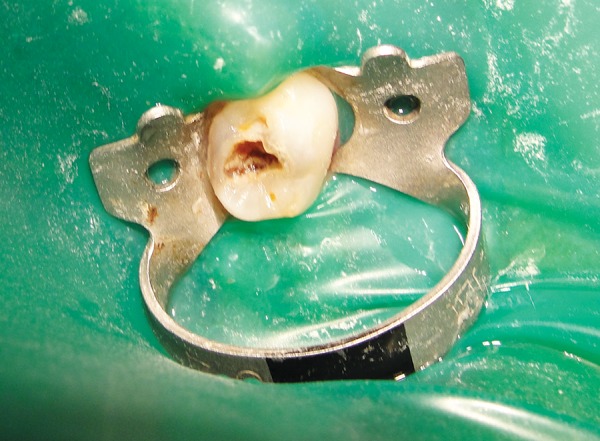
Preoperative photograph showing caries

**Fig. 4:**
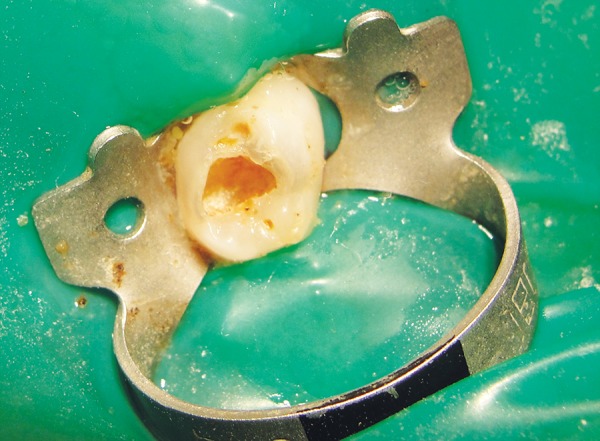
Postoperative photograph showing caries removal using Carisolv

**Fig. 5:**
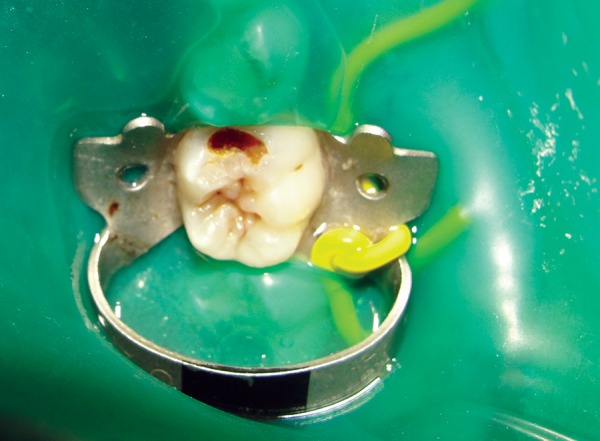
Preoperative photograph showing caries

**Fig. 6:**
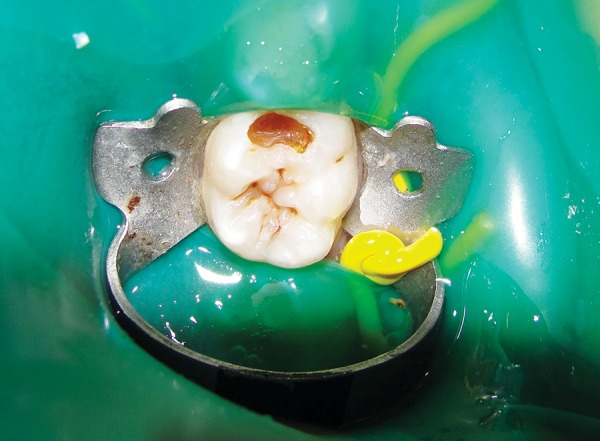
Postoperative photograph showing caries removal using Papacarie

The cases for the present study were selected from the Outpatient Department, Department of Pedodontics and Preventive Dentistry, Sardar Patel Post Graduate Institute of Dental and Medical Sciences, Lucknow. Patients with multiple carious teeth either in the deciduous dentition or mixed dentition were chosen. Ninety primary molars were selected from 30 children (10 males and 20 females) between the age group 6 and 9 years. All children were healthy, without any history of systemic diseases or hereditary anomalies. The study design, objectives, potential benefits and methodology were explained to the selected children and their parents. Consent and ethical committee clearance were obtained prior to the study. The carious teeth were called as samples and were randomly divided into three groups which are as follows:

- *Group I:* Conventional caries removal method using Airotor (Figs 1 and 2).
- *Group II:* Chemomechanical caries removal method using Carisolv (Figs 3 and 4).
- *Group III:* Chemomechanical caries removal method using Papacarie (Figs 5 and 6).

In each group, comprised caries was removed using three different caries removal methods.

### Treatment Evaluation

*Clinical efficacy:* Immediately after the caries excavation, the cavities were evaluated for caries removal or efficacy by the tactile and visual criteria.^5^

**Fig. 7:**
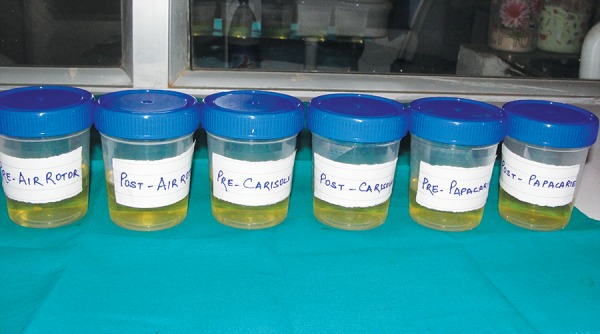
Dentin samples collected in brain heart influsion broth before and after caries removal

*Microbial evaluation:* Before and after the caries removal in each method, the dentin samples were collected with the help of sterile and sharp spoon excavator and immediately transferred to sterile brain heart influsion broth which was used as a transport media for further microbiological investigations (Fig. 7). Minimum two to three visible dentinal scrappings were collected for better microbiological results.

*Microbiological procedure:* The samples collected were placed in an incubator at 37°C for 12 to 24 hours. After 24 hours, media was taken out and screw cap was opened with the help of sterilized bacteriological loop. These samples were then plated on two different Agar plates, i.e. blood agar plates and chocolate agar plates. These plates were incubated at 37°C in anaerobic candle jar for 24 to 48 hours for complete bacterial growth. After the bacterial cultivation the bacterial count was obtained in colony forming units/ml (CFU/ml). For counting the microbial colonies, magnification glass was used. Results were then formulated by the appropriate statistical methods.

*Time taken:* The time taken for all the three procedures were measured from beginning of caries removal till the cavity was confirmed to be free of caries with the help of a stop watch and was recorded in seconds.

*Patient acceptability:* After completion of treatment, patient acceptability toward the treatment was checked with the help of a VAS.^6^

**Table 1:** **Table 1:** Assessment of three groups for clinical efficacy

				*Groups*																	
		*Inference*		*Group I* *(Conventional method)*						*Group II* *(Carisolv)*						*Group III* *(Papacarie)*					
*Score*		*Total no. of teeth = 90*		*No. (30)*				*%*		*No. (30)*				*%*		*No. (30)*				*%*	
0		Complete caries removal from the cavity		20				66.7		0				0		0				0	
1		Caries present at the base of cavity		10				33.3		13				43.3		9				30.0	
2		Caries present at the base and/or one of the walls of cavity		0				0		16				53.3		14				46.7	
3		Caries present in base and/or 2 walls of cavity		0				0		1				3.3		7				23.3	
4		Caries present in base and/or more than 2 walls of cavity		0				0		0				0		0				0	
5		Caries present at base, wall and margins of cavity		0				0		0				0		0				0	

## RESULTS

Table 1 shows the assessment of three groups for clinical efficacy. The efficacy of caries removal had been observed to be the highest with airotor, followed by almost comparable effectiveness by Carisolv and Papacarie method.

The assessment of three groups for microbiological efficacy has been shown in Tables 2 and 3.

On comparing the data using Kruskal-Wallis test (nonparametric ANOVA), no significant difference was observed (p = 0.079) between the three groups.

Table 4 shows the comparison of three study groups for time taken (in seconds) for the procedure.

The mean time taken for procedure in group I was significantly lower as compared to groups II and III. However, statistically, no significant difference was observed between groups II and III as regards the time taken for procedure.

Table 5 shows the comparison of three groups for patient acceptability.

None of the subjects in group I liked the procedure very much. Half of the subjects in group I disliked the procedure very much. In group II, only 1 disliked the procedure, while 11 (36.7%) subjects each liked it a little or liked it very much. In group III 90% liked it very much.

**Table 2:** **Table 2:** Comparison of three groups for microbial load before treatment

				*Groups*																	
		*Inference*		*Group I* *(Conventional method)*						*Group II* *(Carisolv)*						*Group III* *(Papacarie)*					
*Score*		*Total no. of teeth = 90*		*No. (30)*				*%*		*No. (30)*				*%*		*No. (30)*				*%*	
0		No growth		0				0		0				0		0				0	
1		<103		0				0		0				0		0				0	
2		103-104		1				3.3		1				3.3		1				3.3	
3		104-105		11				36.7		16				53.3		14				46.7	
4		Uncountable		18				60		13				43.3		15				50	

**Table 3:** **Table 3:** Comparison of three groups for microbial load after treatment

				*Groups*																	
		*Inference*		*Group I* *(Conventional method)*						*Group II* *(Carisolv)*						*Group III* *(Papacarie)*					
*Score*		*Total no. of teeth = 90*		*No. (30)*				*%*		*No. (30)*				*%*		*No. (30)*				*%*	
0		No growth		10				33.3		4				13.3		4				13.3	
1		<103		17				56.7		22				73.3		19				63.3	
2		103-104		3				10.0		4				13.3		6				20.0	
3		104-105		0				0		0				0		0				0	
4		Uncountable		0				0		0				0		0				0	

## DISCUSSION

The clinical efficacy of group I was higher than groups II and III, both of which had almost comparable efficacy to each other. These results of the present study were in accordance with the studies of Banerjee et al,^7^ Maragakis et al,^8^ Yazici et al,^9^ Peters et al,^10^ who found the similar results when conventional caries removal method was compared to chemomechanical system. The efficacy of removing caries with Airotor was the highest because it tended to over-prepare the cavities because of lack of sensitivity of tactile feedback. This resulted in gross rapid removal of tissue with reduced control over the whole process.

**Table 4:** **Table 4:** Comparison of three groups for time taken (in seconds) for the procedure

*S. no.*		*Groups*		*Mean*		*SD*		*Min.*		*Max.*	
1		I		171.27		23.22		106		200	
2		II		375.33		41.00		300		455	
3		III		387.83		38.53		324		458	

**Table 5:** **Table 5:** Comparison of three groups for patient acceptability

				*Groups*																	
		*Inference*		*Group I* *(Conventional method)*						*Group II* *(Carisolv)*						*Group III* *(Papacarie)*					
*Score*		*Total no. of teeth = 90*		*No. (30)*				*%*		*No. (30)*				*%*		*No. (30)*				*%*	
1		Dislike very much		15				50		0				0		0				0	
2		Dislike a little		14				46.7		1				3.3		0				0	
3		Not sure		1				3.3		7				23.3		0				0	
4		Like a little		0				0		11				36.7		3				10	
5		Like very much		0				0		11				36.7		27				90	

But, few other studies by Ericson et al^5^ and Fureet al ^11^ concluded almost comparable clinical efficacy of conventional and chemomechanical caries removal systems.

The microbiological results of the present study revealed after the procedure, majority of subjects in all the three groups had microbial colony count <103. 33.3% teeth in group I showed no microbial growth followed by 13.3% each in groups II and III. Azrak et al,^12^ Sterer et al^13^ and Subramaniam et al^14^ reported similar results as the present study, microbiological efficacy of chemomechanical caries removal method was comparable with that of conventional method.

As reviewed by various researchers, the antimicrobial property of Carisolv has been attributed to sodium hypo-chlorite, its main constituent, which is effective against bacteria in dental infections and cariogenic bacteria. They have reported that sodium hypochlorite causes biosyn-thetic alteration in cellular metabolism, phospholipid destruction and formation of chloramines interferes in the cellular metabolism and causes irreversible enzyme activation.

According to Dawkins et al,^15^ Bu ssadori et al^3^ and Motta et al,^16^ papain in Papacarie gel is a proteo-lytic enzyme with bactericidal, bacteriostatic and anti-Inflammatory characteristics. It also acts as a debridant which does not damages healthy tissue and accelerates the cicatricial process.

In the present study, the mean time taken for procedure in group I was 171.27 ± 23.22 seconds which was significantly lower as compared to group II (375.33 ± 41 seconds) and group III (387.83 ± 38.53 seconds) respectively.

Similar study conducted by Jawa et al^17^ in dicated that mean time for complete caries excavation with chemo-mechanical method using Papacarie was 328.5 seconds as compared to that of 124.6 seconds with convention caries excavation method which was in accordance with present study. Other studies in confirmation with present study were by Ericson et al,^5^ Banerjee et al , ^7^ Ansari et al ,^18^ Rafique et al,^19^ Yazici et al^9^ and Jawa et al.^17^ Their results suggested that the mean time taken in caries excavation by chemomechanical method was significantly higher as compared to that by conventional method.

After completion of treatment, patient acceptability toward the treatment was checked with the help of a VAS (five-point facial hedonic scale). The scale was graded according to the patient accept a nice toward the procedure. Point 1 denotes that patient disliked the procedure very much to point 5 denotes that patient liked the procedure very much.

In the present study, 15 out of 30 subjects in group I disliked the procedure very much (score 1), 14 (46.7%) disliked it a little (score 2), while remaining 1 (3.3%) was not sure. In group II, only 1 out of 30 (3.3%) disliked the procedure, 7 (23.3%) were not sure while 11 (36.7%) subjects each liked it a little or liked it very much. In group III, only 3 (10%) subjects liked the procedure a little while the remaining 27 out of 30 (90%) liked it very much.

Other studies conducted by Rafique et al^19^ Lozano-Chourio et al^20^ and Pandit et al^21^ showed the similar results concluding that chemomechanical caries removal (CMCR) method was more acceptable than conventional drilling method.

## CONCLUSION

- The clinical efficacy of caries removal was highest with Airotor followed by almost comparable effectiveness by Carisolv and Papacarie.
- The microbiological efficacy of caries removal was almost comparable with Airotor, Carisolv and Papacarie methods.
- The time taken to remove caries by Airotor method was observed to be significantly lower as compared to that taken by Carisolv and Papacarie. Carisolv and Papacarie had almost comparable values of time taken.
- Patient acceptance during caries removal was found to be highest with Papacarie method followed by Carisolv and least by Airotor method.

Thus, it was concluded from the study that even though Papacarie and Carisolv were time-consuming methods, they removed caries effectively and with high patient acceptance and, therefore, they can be considered as viable alternatives to painful caries removal technique like Airotor in the management of dental caries, especially in pediatric patients.
